# Ameliorative Effects of Component Chinese Medicine From *Curcumae Rhizoma* and *Sparganii Rhizoma*, a Traditional Herb Pair, on Uterine Leiomyoma in a Rat Model

**DOI:** 10.3389/fpubh.2021.674357

**Published:** 2021-05-28

**Authors:** Li Zhang, Qiuxia Xu, Yao Li, Hui Zhao, Xingming Shi, Fu Peng, Chenghao Yu

**Affiliations:** ^1^Basic Medicine College, Chengdu University of Traditional Chinese Medicine, Chengdu, China; ^2^Jianghai Branch, Jiangmen Central Hospital, Guangdong, China; ^3^Department of Pediatrics, The Second Hospital Affifiliated Shaanxi University of Chinese Medicine, Shaanxi, China; ^4^Institute of Basic Theory, China Academy of Chinese Medical Sciences, Beijing, China; ^5^Medical College of Georgia, Augusta University, Augusta, GA, United States; ^6^West China School of Pharmacy, Sichuan University, Sichuan, China; ^7^State Key Laboratory of Southwestern Chinese Medicine Resources, Sichuan, China

**Keywords:** uterine leiomyoma, *Curcumae Rhizoma*, *Sparganii Rhizoma*, estrogen, progesterone, hemorheology, mechanism study

## Abstract

Uterine leiomyoma (UL), common benign tumors in women of child-bearing age, are believed to be caused mainly by Qi stagnation and blood stasis, according to a theory of traditional Chinese medicine. *Curcumae Rhizoma* and *Sparganii Rhizoma* (CRSR) is a classical herb pair that activates blood circulation to dissipate blood stasis. The purpose of this study was to explore the prevention and treatment effects of CRSR component compatibility on UL in rats. We randomly assigned adult female non-pregnant rats into three groups: a normal control (NC) group, a UL model group, and a CRSR treatment group. We administered to the UL and CRSR groups oral gavage diethylstilbestrol and injected them with progesterone (P) to establish UL for 5 weeks. The CRSR group received a CRSR medicinal solution after daily modeling. The uterus morphology of the UL group showed significantly more swelling than did that of the NC group, and we found no significant abnormalities in the morphology of the CRSR group. The pathological changes associated with UL were relieved in the CRSR group. CRSR improved the related parameters of the uterus and ovarian coefficients, significantly reducing the concentrations of P in the serum and the concentrations of estradiol, P, estrogen receptor, and P receptor in the uterus and ovary. In addition, CRSR significantly improved the abnormal blood conditions of UL, shown by decreases in plasma viscosity, the erythrocyte sedimentation rate equation *K* value, and erythrocyte aggregation index. Therefore, CRSR component compatibility may prevent and cure UL through the above ways.

## Introduction

Uterine leiomyoma (UL), or fibroids, are the most common tumors in women, caused by the proliferation of smooth muscle cells. Because of the difference in diagnostic methods and study participants, the prevalence rate varies from 4.5 to 68.6%, and the number of Black women with UL far exceeds that of white women (1). Although UL tumors are benign, they can cause symptoms such as dysmenorrhea, abnormal uterine bleeding, pelvic pain, and recurrent abortion, all of which may require medicine or surgery (2, 3).

UL are recognized as a hormone-dependent tumor, estrogen, progesterone (P), and their receptors can play an important role in its occurrence and development (3–5). Estradiol (E_2_) can induce the expression of the progesterone receptor (PR) to directly affect human leiomyoma cells (6). P can promote the proliferation and complete development of leiomyoma cells (5). Thus, the current medical treatment of UL mainly is inhibiting steroid hormones (e.g., antiprogestins, aromatase inhibitors), but these can only reduce the size of a tumor, and after discontinuing use, the tumor will continue to grow (6). Although surgical resection is a relatively thorough method, it is not suitable for patients seeking to preserve their uterus or fertility, and the costs associated with surgical treatment can be an economic burden (7). Therefore, alternative economic and effective non-surgical treatment methods are needed.

Traditional Chinese medicine (TCM) has been used widely in the treatment of UL (8–10). Studies have shown that the surgical rate of patients with UL treated with TCM is significantly reduced (8), as are medication dosage and total medical expenses, as compared to conventional Western medicine (9). In TCM theory, UL is believed to be caused by stagnation of Qi and blood in the pelvic region over a certain time period (11). The prevention and treatment of hysteromyoma using TCM can normalize blood flow and unobstruct Qi. If Qi and blood do not accumulate in the uterus, tumors will not produce, so the treatment of UL mostly adopts the TCM formula of promoting blood circulation and removing blood stasis (10, 11).

Compare with TCM formula, herbal pairs, as the most basic form of TCM, can not only reflect the characteristics of TCM but also have simple ingredients, which is convenient for research (12). In general, a herbal pair is composed of two types of herbs.

CRSR, a classical herb pair used to promote blood circulation and remove blood stasis, is commonly used in the treatment of gynecological tumors (13–16). *Curcumae Rhizoma* has antitumor, antiplatelet aggregation, antithrombosis, hepatoprotective, antimicrobial, and other pharmaceutical activities. *Curcumae Rhizoma* essential oil also is widely used in China for the treatment of tumors (16). *Sparganii Rhizoma* has anti-estrogen, anticancer, and anti-angiogenic effects (14, 17). *Sparganii Rhizoma* can combine with *Curcumae Rhizoma* through its own antitumor activity compound (linear diarylheptanoids) to enhance the efficacy of CRSR (13). Xu et al. (15) also has found that the volatile oil of CRSR showed stronger antioxidant and anticancer activity, and its inhibitory effect on tumor cells was more significant than that of *Curcumae Rhizoma* oil or *Sparganii Rhizoma* oil alone.

Although CRSR has shown good effects on UL in clinical practice and experimental research, few have studied its prevention and treatment effects and its mechanisms of component compatibility. Therefore, in this study, we took CRSR intervention while making a UL model, from organ coefficients (ovary, uterus), histopathology, sex hormone levels (E_2_, P, and their receptors), and hemorheology to explore the prevention and treatment effects of CRSR on UL.

## Materials and Methods

### Reagents and Extract Preparation

*Sparganii Rhizoma* slices were obtained from Sichuan Hao-Bo Co., Ltd. (Lot no. 160301; China). Volatile oil of *Curcumae Rhizoma* was obtained from Shanxi Hao-Chen Biotechnology Co., Ltd. (Lot no. EZ150801; China). Diethylstilbestrol was provided by Chengdu West Asia Chemical Co., Ltd. (Lot no. K9212; China). P injections were manufactured by Zhejiang Xian-Ju Pharmaceutical Co., Ltd. (Lot no. 150610; China). Hematoxylin was purchased from Beijing Bailingwei Technology Co., Ltd. (Lot no. LM10N13; China). Eosin dye liquor was produced by Tokyo Chemical Industry Co., Ltd. (Lot no. GL01-GMPC; Japan).

ELISA kits for Rat E_2_ (Lot no. XL-Er0478), P (Lot no. XL-Er0477), ER (Lot no. XL-Er1021), and PR (Lot no. XL-Er0474) were produced by Aimejie Technology Co., Ltd., and imported by Beijing Yonghui Biotechnology Co., Ltd., China.

*Sparganii Rhizoma* was smashed into coarse powder and passed through a no. 24 mesh sieve. The powder was soaked in 10 × volume of 60% ethanol for 30 min. The reflux extraction was carried out twice by heating with an electric heating jacket for 1.5 h each time. After filtration with a vacuum pump, alcohol was recovered by decompression with a rotary evaporator, and the collected concentrated liquid was degreased with petroleum ether. Finally, polyamide column chromatography was performed. After chromatography, distilled water was used for elution, ethanol was used for elution and alcohol recovery. After placement in a constant-temperature drying oven, a solution of 1 g/mL was prepared with distilled water and stored at 4°C.

The volatile oil of *Curcumae Rhizoma* was emulsified with 1% tween −80, and a solution of 1 g/ml was prepared with distilled water. These two solutions were mixed at 1:1, configured as a CRSR solution, and stored at 4°C.

### Construction of a UL Model in Rats and CRSR Treatment

All animal experimental protocols were reviewed and approved by the Animal Ethics Committee of Chengdu University of TCM. We obtained 27 non-pregnant female adult Sprague–Dawley rats (200 ± 20 g) from Jianyang Dashuo Experimental Animal Tech (Sichuan, China) with a license number SCXK (Chuan) 2013-24. Rats were kept in the Center of Laboratory Animals at Chengdu University of TCM. After 5 days of adaptive feeding, we divided the rats randomly into a normal control (NC) group (*n* = 9), a UL group (*n* = 9), and a CRSR group (*n* = 9).

We extracted the UL modeling method using our group's previous research (16). The UL and CRSR groups received a 1.35 mg/kg daily gavage of diethylstilbestrol, and 1.0 mg of progesterone intramuscularly injected every other day for 5 weeks. The CRSR group received medicine formulated from *Curcumae Rhizoma* and *Sparganii Rhizoma* (6.67 g/kg) 2 h after daily intragastric administration of the diethylstilbestrol. Meanwhile, the NC group received 2 ml of distilled water every day and 0.05 ml of normal saline injected intramuscularly every other day. We monitored the diet, weight, and general behavior of the rats weekly.

### Sample Harvest

At the end of the experiment, the rats were sacrificed after an overnight fast, the rats were anesthetized by 10% chloral hydrate. We collected the blood samples (5 ml each) in vacutainer tubes from the femoral artery. The blood was allowed to clot before centrifugation at 3,500 rpm for 10 min at 6°C and then stored at −80°C until analysis. We peeled off the uterus and ovaries, removed the adipose tissue on the surface, and removed the blood stains with saline and then weighed and measured these separately. We measured the transverse diameter *(r)* and diameter *(R)* with vernier calipers. We calculated the uterine and ovarian coefficients using the following formula: coefficient = single organ weight (mg)/final body weight (g) ×100%. After taking the measurements, we stored half of the uteruses and ovaries in 4% paraformaldehyde fixative and the other half in liquid nitrogen.

### Uterine Histopathologic Examination

We processed and stained the fixed uterine tissues with hematoxylin and eosin. The section images were acquired with a digital photographic microscope (BA400Digital; Mike Audi Industry Group Co., Ltd.).

### Enzyme-Linked Immunosorbent Assay

ELISA kits determined the concentrations of serum E_2_ and P, as well as the levels of E_2_, P, ER, and PR in the uterine and ovarian homogenate tissues following the manufacturer's instructions.

### Determination of Hemorheology

We determined the plasma viscosity (PV), the erythrocyte sedimentation rate equation *K*'s value (*K* value of ESR equation), and the erythrocyte aggregation index (EAI) in blood samples with an automatic blood rheometer (Sa-5600; Beijing Seksid Technology Development Co., Ltd.).

### Statistical Analysis

All data were statistically analyzed by SPSS 21.0, reporting the results as mean ± SD. We used one-way analysis of variance (ANOVA) and the least significant difference (LSD) test if the variances are homogeneous; otherwise, used the Kruskal–Wallis H test. We considered *p* < 0.05 as statistically significant.

## Results

### Observation of the Rats' General Behavior

Before the establishment of the models, all rats ate and drank normally, had white and glossy fur, and were in good physical and mental health. After we established the UL model, the rats developed mental atrophy, and their fur became yellow and had severe depilation. In the later stage of modeling, the rats were easily irritated and fought.

### CRSR Reduced Uterine Swelling in the UL Rats

**Figure 1:**
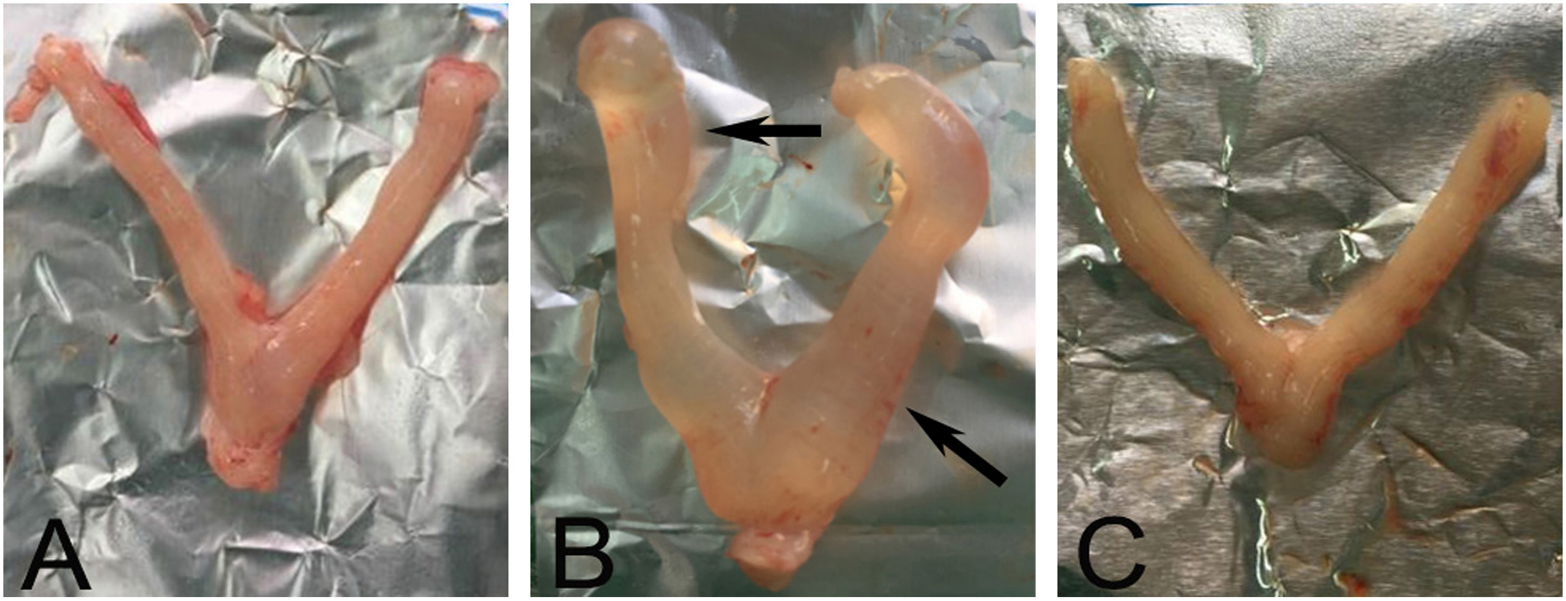
Representative images of morphology of a rat uterus after CRSR treatment. The uterus of a NC rat **(A)** was bilaterally symmetrical, without swelling nodules and cysts. Significant swelling, nodules, and cysts (arrows) appeared in the UL group **(B)**. The CRSR uterus morphology **(C)** tended to match that of the NC group **(A)**. NC, normal control group; UL, uterine leiomyoma model group; CRSR, *Curcumae Rhizoma* and *Sparganii Rhizoma* treatment group.

The uteruses of the rats in the NC group were bilaterally symmetrical and y shaped, with uniform texture; bright color; and no cysts, nodules, or swelling (Figure 1A). The length and thickness of the uteruses in the UC group were uneven, with faded color and obvious swelling, nodules, and cysts (Figure 1B). The shape of the uteruses in the CRSR group were symmetrical, their volume was greatly reduced, and their color became pale (Figure 1C), we observed no swelling, cysts, or nodules. The UL modeling method resulted in swelling, nodules, and cysts. After CRSR treatment, uterine morphology was significantly improved and tended to be normal (Figure 1A).

### Histology Analysis

**Figure 2:**
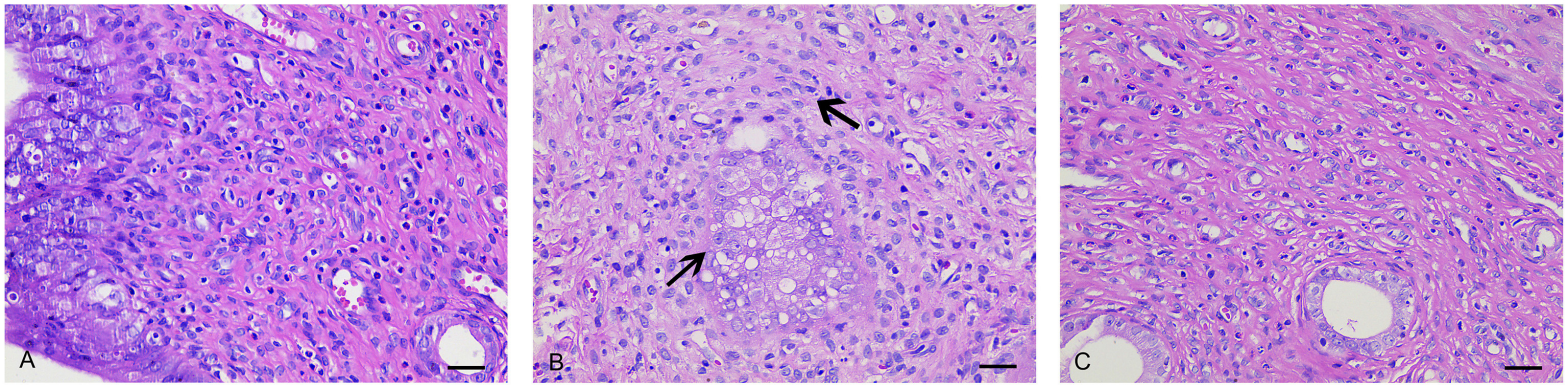
Histopathologic examinations of uteruses from the NC, UL, and CRSR groups. The original magnification was 400×. Scale bar: 30 μm. **(A)** NC, normal control group; **(B)** UL, uterine leiomyoma model group; **(C)** CRSR, *Curcumae Rhizoma* and *Sparganii Rhizoma* treatment group.

In the NC group (Figure 2A), the endometrium was intact, with no signs of pathological changes such as hyperplasia, atrophy, hyperemia, edema, denaturation, necrosis, or inflammatory cell infiltration. The epithelial cells were aligned, with no thickening or shedding degeneration. The volume of the smooth muscle cells and serosal layer were normal. In the UL group (Figure 2B), the uterine smooth muscle cells showed hyaline degeneration and focal hyperplasia, and the mucosal epithelial cells were vacuolated. The uterine smooth muscles were slightly hypertrophic. After CRSR treatment (Figure 2C), the endometrium was largely intact, with only a small amount of lymphocyte infiltration in the lamina propria. These histological changes further demonstrated the preventive and therapeutic effects of CRSR on UL.

### CRSR Improved the *r* and *R* of the Uterus

**Figure 3:**
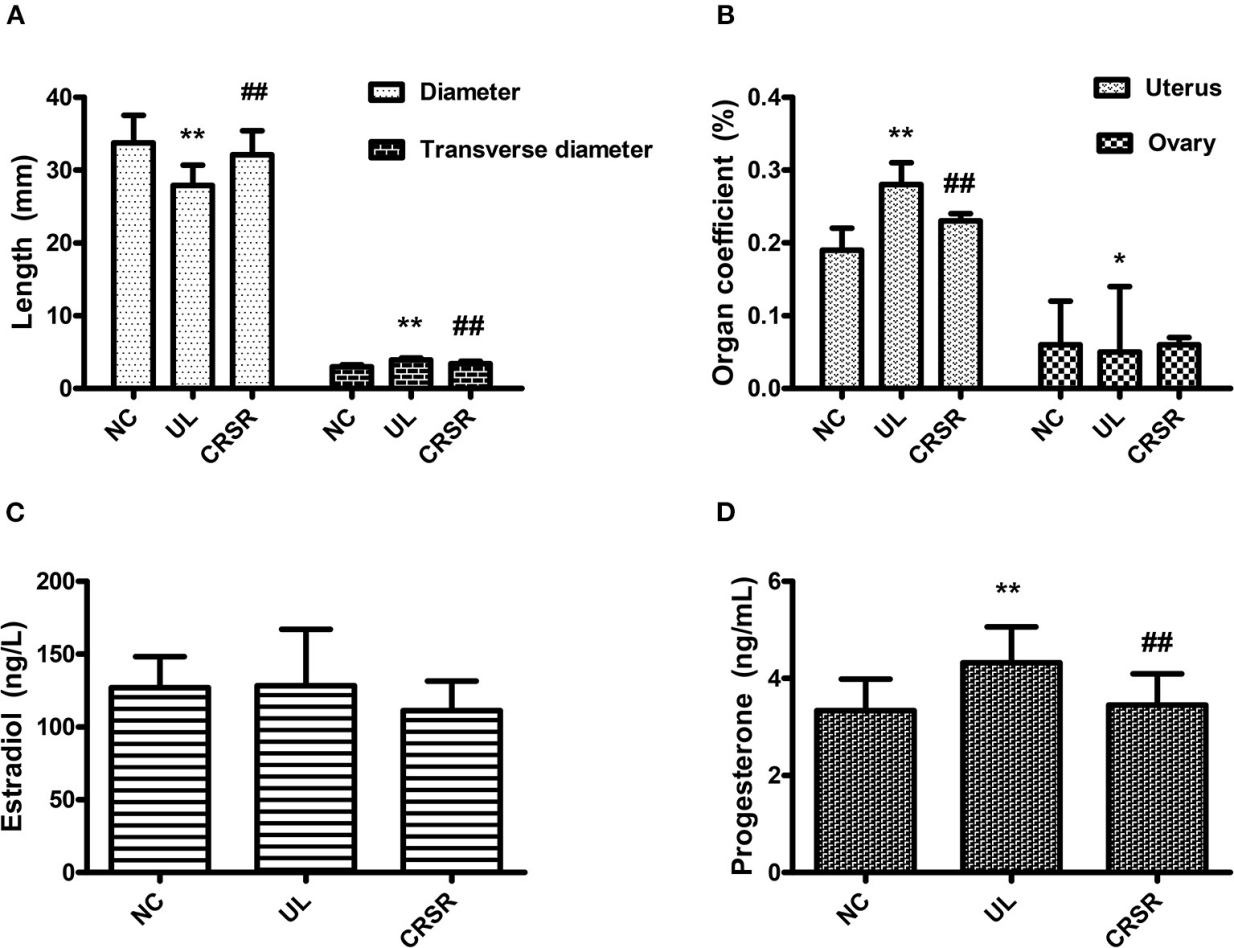
**(A)** Effects of CRSR on the transverse diameter and diameter of the uterus. **(B)** Effects on the uterine and ovarian coefficients. **(C,D)** Changes of E_2_ and P in the serum. Data are reported as mean ± SD. NC, normal control group; UL, uterine leiomyoma model group; CRSR, *Curcumae Rhizoma* and *Sparganii Rhizoma* treatment group; E_2_, estradiol; P, progesterone. *p < 0.05, **p < 0.01 UL vs. NC; ^#^*p* < 0.05, ^*##*^*p* < 0.01 CRSR vs. UL (one-way ANOVA and LSD test).

The uterine *r* in the UL group was significantly wider than that in the NC group, and the uterine *R* was significantly shorter than that in the NC group (*p* < 0.01). The *r* of the uteruses in the CRSR group was significantly shorter than that in the UL group, and the *R* was significantly increased, with a value close to that in NC group (*p* < 0.01) (Figure 3A).

### Effects of CRSR on the Uterus and Ovary Coefficients in the UL Rats

The uterine coefficient increased significantly in the UL group as compared with the NC group (*p* < 0.01). In contrast, the uterine coefficient decreased significantly in the CRSR group as compared with the UL group (*p* < 0.01). As compared with the NC group, the ovarian organ coefficient of the UL group significantly decreased (*p* < 0.05). The ovarian organ coefficient of the CRSR group showed a slight increase as compared with the UL group, but the difference was not statistically significant (*p* > 0.05) (Figure 3B).

### CRSR Reduced Serum E_2_ and P Concentrations in the UL Rats

We observed no significant differences in the concentrations of E_2_ among the three groups (*p* > 0.05) (Figure 3C). The concentrations of P in the UL group's serum was significantly higher than that in the NC group (*p* < 0.01). The concentrations of P in the CRSR group's serum as significantly reduced as compared with that in the UL group (*p* < 0.01) (Figure 3D).

### CRSR Significantly Reduced the Levels of E_2_, P, ER, and PR in Uterine and Ovarian Tissues

**Figure 4:**
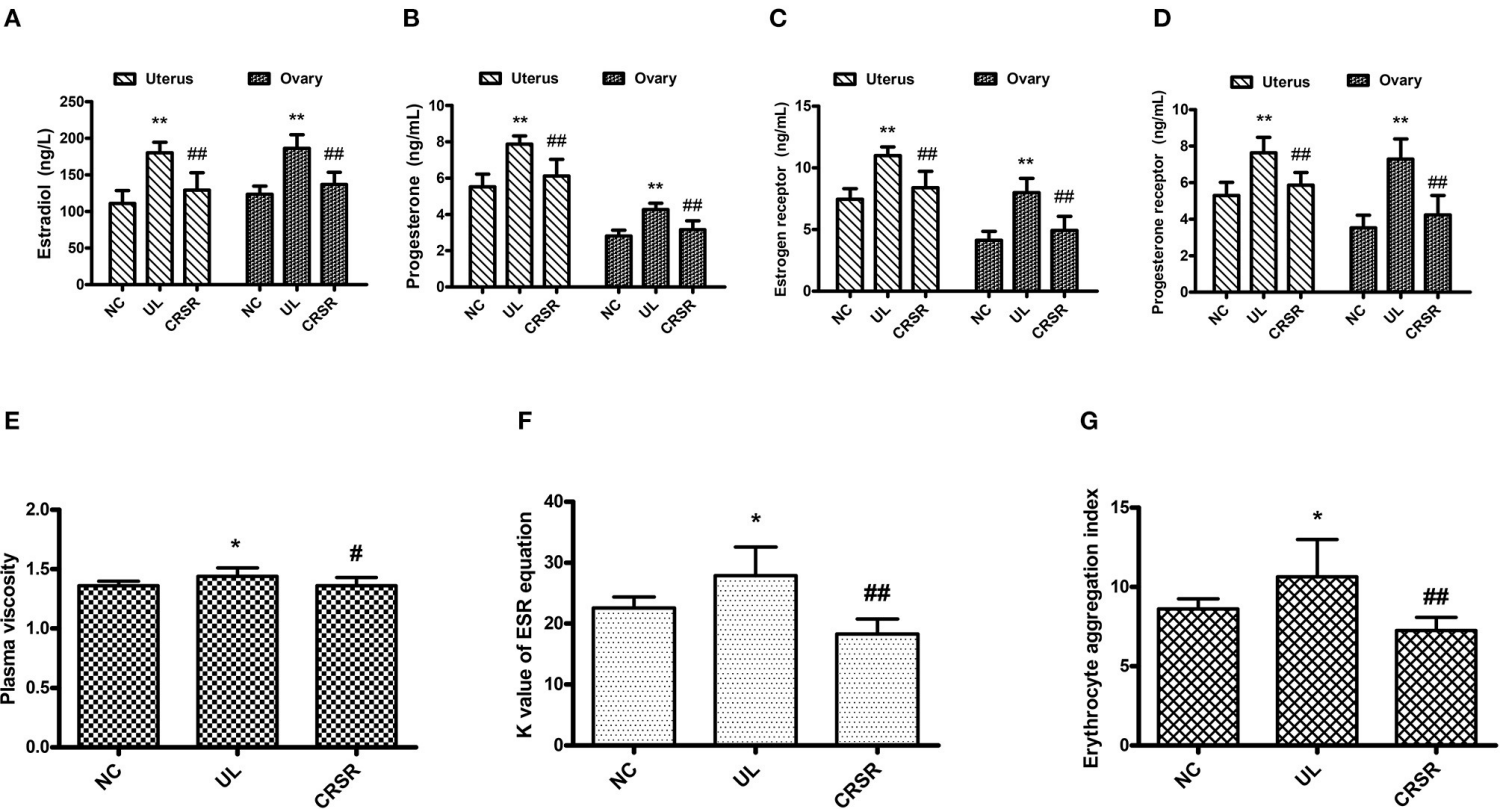
CRSR reduced the levels of key hormones and receptors of UL. **(A)**, estradiol (E_2_); **(B)**, progesterone (P); **(C)**, estrogen receptor (ER); **(D)**, progesterone receptor (PR). **(E–G)** Effects of CRSR on hemorheology. **(E)**, PV; **(F)**, K value of ESR equation; **(G)**, EAI. Data are reported as mean ± SD. NC, normal control group; UL, uterine leiomyoma model group; CRSR, *Curcumae Rhizoma* and *Sparganii Rhizoma* treatment group. *p < 0.05, **p < 0.01 UL vs. NC; ^#^*p* < 0.05, ^*##*^*p* < 0.01 CRSR vs. UL (Kruskal–Wallis H test).

As compared with the NC group, the levels of E_2_, P, ER, and PR in the uteruses and ovaries in the UL group were significantly increased (*p* < 0.01). The concentrations of E_2_, P, ER, and PR in the uteruses and ovaries of the CRSR group were significantly decreased as compared with the UL group (*p* < 0.01) (Figures 4A–D).

### CRSR Restored Abnormal Hemorheology

We assayed the PV, *K* value of ESR equation and EAI in the blood samples of all groups. Data collected from each group were not homogeneous by test of variance. Therefore, the Kruskal–Wallis H test was adopted.

The PV, *K* value of ESR equation, and EAI in the UL group increased significantly as compared with those in the NC group (*p* < 0.05). As compared with the UL group, the PV of the CRSR group was significantly lower (*p* < 0.05), and the *K* value of ESR equation and EAI were significantly reduced (*p* < 0.01) (Figures 4E–G).

## Discussion

UL is a distressing disease for women around the world (1, 2, 18), not only because it seriously affects their physical health but also because it affects their mental health. Women with UL usually feel more depressed, irritable, and uneasy than do women without UL and can have a higher risk of depression (19, 20). Effective prevention and treatment of UL can reduce women's health burden and improve their quality of life. In clinical practice, TCM treatment of UL has a better overall effect and fewer side effects and other advantages (8, 10, 15).

In our study, the uteruses of the UL group showed obvious swelling and nodules, and the uteruses morphology of the CRSR group tended to be more normally. With the intervention of CRSR, the volume of the uteruses was greatly reduced. The uterine *r* and *R*, as well as the uterus and ovary coefficients, were improved. In addition, the decrease of pathological changes in the uterine tissues showed that CRSR can inhibit the growth of UL.

E_2_ and P are the key factors in tumor development, stimulating the mature leiomyoma cells in UL to produce mitogenic substances to promote the proliferation of immature cells (5). UL pathology is also related to some abnormalities of ER and PR and the signaling pathways that they mediate (4, 21). In clinical trials, selective ER and PR modulators can effectively inhibit the growth of UL (5). Therefore, controlling the expression of estrogen and P and their receptors is the primary way to prevent and treat UL.

Through the detection of E_2_, P, ER and PR, we found that CRSR can reduce the high expression of E_2_, P, ER, and PR in the UL rats. Reduced the concentration of P and E_2_ in the serum, especially the concentration of P. The concentration of E_2_, P, and their receptors in ovarian and uterine tissues were significantly reduced and tended to be at the levels of the NC group, indicating the pharmacological effects of CRSR (14, 17). The significant changes in the serum, uteruses, and ovaries suggest that CRSR not only targets one location but also has multiple targets.

From the perspective of TCM, blood stasis is an important reason for the formation of UL. The pathogenesis of blood stasis is related to abnormal hemorheology, mainly involving whole blood viscosity, PV, ESR equation, and erythrocyte aggregation (22–25). PV is one of the important factors affecting the viscosity of whole blood. Increased PV will inevitably increase whole blood viscosity. Red blood cells (RBCs) account for nearly 50% of blood volume and are the main component of the cell content in the blood. Both ESR and EAI can reflect the changes of RBCs.

To explore whether the prevention and treatment of UL by CRSR is related to the improvement of congestion, we compared the PV, *K* value of ESR equation, and EAI of the rats from each group. As compared with the NC group, the PV, *K* value of ESR equation, and EAI of the UL group were significantly increased, indicating that blood viscosity increased, erythrocyte aggregation increased, blood flow decreased, microcirculation deteriorated, and blood stasis syndrome existed. These results are consistent with the understanding of UL pathogenesis in TCM theory. TCM can remove blood stasis by activating blood circulation (23–25). The antitumor and antithrombotic effects of *Curcumae Rhizoma* are related to activating blood circulation to dissipate blood stasis (16). The significant decrease of PV, *K* value of ESR equation, and EAI in the CRSR group suggests that CRSR can reduce plasma viscosity, inhibit erythrocyte aggregation, and restore abnormal hemorheology, proving the efficacy of CRSR in promoting blood circulation and removing blood stasis (15, 16, 23, 24). CRSR inhibition of UL growth is related to the improvement of hemorheology.

Our study has proved that CRSR has positive prevention and treatment effects on UL, which is achieved by reducing uterine volume, changing uterine structure, regulating hormone levels, and restoring abnormal blood circulation disorders. Our work provides a new basis for the prevention and treatment of UL for CRSR.

## Data Availability Statement

The raw data supporting the conclusions of this article will be made available by the authors, without undue reservation.

## Ethics Statement

The animal study was reviewed and approved by the Animal Ethics Committee of Chengdu University of Traditional Chinese Medicine.

## Author Contributions

FP and CY conceived and designed the experiment. QX, YL, and HZ carried out the experiment and analyzed the experimental data. LZ retrieved the relevant literature and wrote the manuscript. XS provided helpful comments and revised the manuscript. All authors contributed to the article and approved the submitted version.

## Conflict of Interest

The authors declare that the research was conducted in the absence of any commercial or financial relationships that could be construed as a potential conflict of interest.
